# Advancing Clinical Chatbot Validation Using AI-Powered Evaluation With a New 3-Bot Evaluation System: Instrument Validation Study

**DOI:** 10.2196/63058

**Published:** 2025-02-27

**Authors:** Seungheon Choo, Suyoung Yoo, Kumiko Endo, Bao Truong, Meong Hi Son

**Affiliations:** 1 Research Institute for Future Medicine, Samsung Medical Center, Seoul, Republic of Korea; 2 Department of Digital Health, Samsung Advanced Institute for Health Sciences and Technology (SAIHST), Sungkyunkwan University, Seoul, Republic of Korea; 3 Med2Lab Inc, San Francisco, CA, United States; 4 Department of Emergency Medicine, Samsung Medical Center, Seoul, Republic of Korea

**Keywords:** artificial intelligence, patient education, therapy, computer-assisted, computer, understandable, accurate, understandability, automation, chatbots, bots, conversational agents, emotions, emotional, depression, depressive, anxiety, anxious, nervous, nervousness, empathy, empathetic, communication, interactions, frustrated, frustration, relationships

## Abstract

**Background:**

The health care sector faces a projected shortfall of 10 million workers by 2030. Artificial intelligence (AI) automation in areas such as patient education and initial therapy screening presents a strategic response to mitigate this shortage and reallocate medical staff to higher-priority tasks. However, current methods of evaluating early-stage health care AI chatbots are highly limited due to safety concerns and the amount of time and effort that goes into evaluating them.

**Objective:**

This study introduces a novel 3-bot method for efficiently testing and validating early-stage AI health care provider chatbots. To extensively test AI provider chatbots without involving real patients or researchers, various AI patient bots and an evaluator bot were developed.

**Methods:**

Provider bots interacted with AI patient bots embodying frustrated, anxious, or depressed personas. An evaluator bot reviewed interaction transcripts based on specific criteria. Human experts then reviewed each interaction transcript, and the evaluator bot’s results were compared to human evaluation results to ensure accuracy.

**Results:**

The patient-education bot’s evaluations by the AI evaluator and the human evaluator were nearly identical, with minimal variance, limiting the opportunity for further analysis. The screening bot’s evaluations also yielded similar results between the AI evaluator and human evaluator. Statistical analysis confirmed the reliability and accuracy of the AI evaluations.

**Conclusions:**

The innovative evaluation method ensures a safe, adaptable, and effective means to test and refine early versions of health care provider chatbots without risking patient safety or investing excessive researcher time and effort. Our patient-education evaluator bots could have benefitted from larger evaluation criteria, as we had extremely similar results from the AI and human evaluators, which could have arisen because of the small number of evaluation criteria. We were limited in the amount of prompting we could input into each bot due to the practical consideration that response time increases with larger and larger prompts. In the future, using techniques such as retrieval augmented generation will allow the system to receive more information and become more specific and accurate in evaluating the chatbots. This evaluation method will allow for rapid testing and validation of health care chatbots to automate basic medical tasks, freeing providers to address more complex tasks.

## Introduction

Faced with a projected shortfall of 10 million health care workers by 2030 [1], the health care sector urgently requires innovative solutions to sustain patient care and education. Artificial intelligence (AI) automation in low- to mid-level tasks like patient education and initial therapy screening emerges as a strategic response to mitigate this shortage, reallocating medical staff to higher-priority tasks [2,3].

The advent of advanced multimodal large language models (LLMs) such as GPT-4 introduces a paradigm shift, promising scalable, cost-effective chatbot solutions, which are particularly helpful for tasks that require the provider to interact with the patient [4]. GPT-4 and similar models offer a more dynamic, conversational approach, tailoring information to individual patient needs with minimal logistical or financial overhead for health care institutions. This technological evolution promises not only to fill the imminent workforce gap but also to enhance the quality and accessibility of health care services, leveraging AI’s capacity for on-demand, personalized patient support [4-7]. It has been reported that LLMs have the cognitive capacity to role-play the character as portrayed in the dialogue prompt [8]. Shao et al [9] showed that GPT-3.5 can be used to score the believability of LLM role-playing. Finally, Yang et al [10] pointed to the high potential that medical chatbots have in clinical settings, while Gilbert et al [11] warned of the need to extensively test health care chatbots.

However, current methods of creating and evaluating early-stage health care bots face steep development costs due to the high level of human involvement in each phase of the development process. In this study, we present a novel, bot-driven method of developing, testing, and evaluating automated health care chatbots. At the center of this strategy is the use of the LLM as an “evaluator agent” to iteratively review and provide feedback on the dialog between the health care bot being evaluated and a set of “digitally simulated patients” also role-played by the LLM. This approach provides a fully automated system that will not only reduce the amount of time and effort required to develop the chatbots but also provide a feasible way to continuously monitor the performances of health care chatbots in different clinical settings.

## Methods

### Study Design

**Figure 1.**
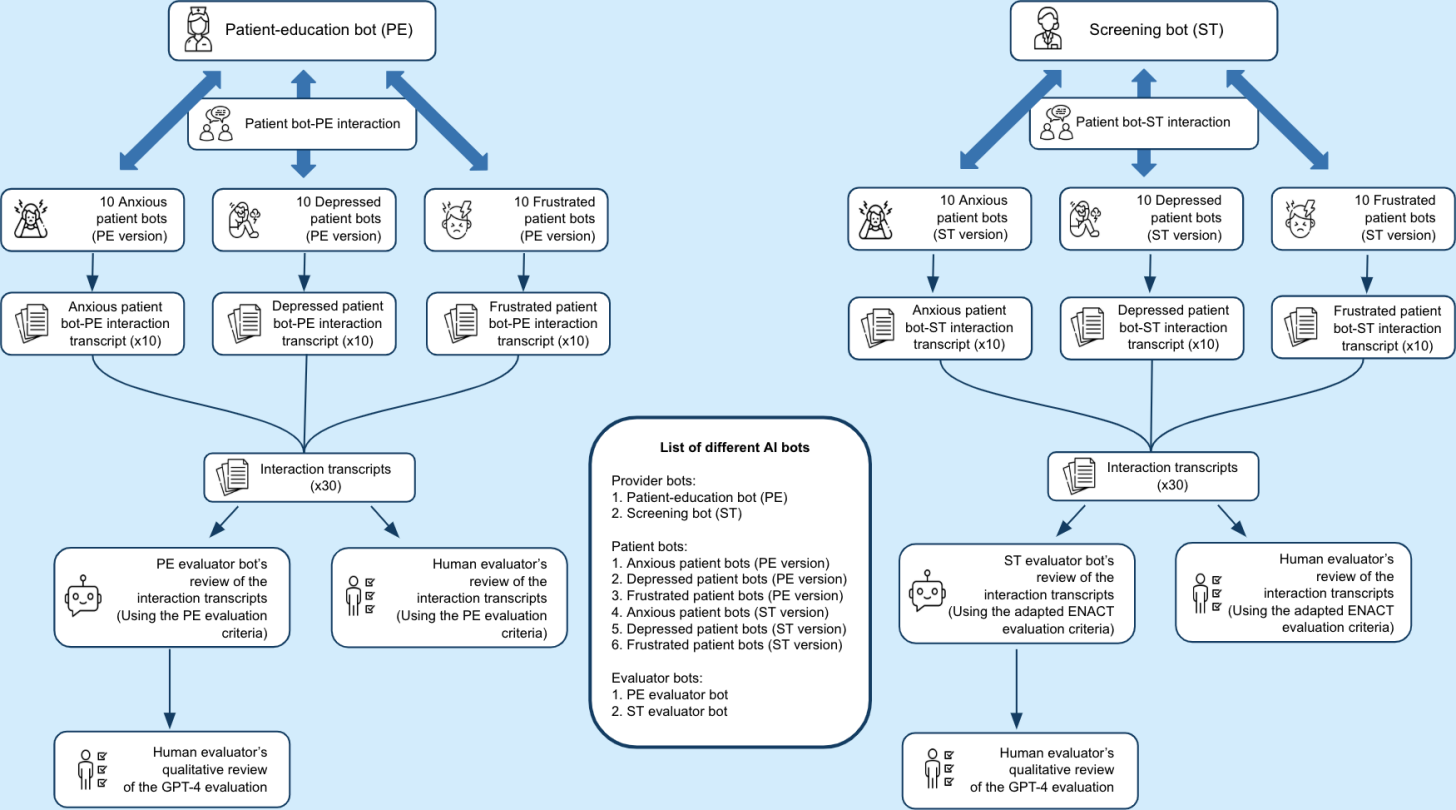
Interaction and evaluation methodology for the patient-education bot and the initial screening bot. Includes a list of all the different bots used. AI: artificial intelligence; ENACT: Enhancing Assessment of Common Therapeutic Factors.

This study introduces a novel bot-driven method to evaluate the abilities of LLMs in health care tasks. In this approach, LLMs were configured to perform as a patient-education bot, a pretherapy screening bot, patient bots, and evaluator bots. The patient bots simulated distinct emotional personas—depressed, anxious, and frustrated—to test the adaptability and competency of the provider bots. The evaluator bots assessed the interactions based on predefined criteria. Results from the AI evaluations were cross-referenced with human expert reviews for accuracy and reliability (Figure 1).

### Setup

To demonstrate the system, 2 AI provider bots were developed using GPT-4 in collaboration with an experienced oncology nurse and a licensed cognitive behavior counselor. One provider bot emulated a patient-education nurse, delivering medical information with clarity and empathy. The second bot acted as a mental health therapist, modeled on acceptance and commitment therapy and mindfulness practices, to provide nonpharmacological mental health support.

AI patient bots, also developed using GPT-4, were programmed to represent 40-year-old male patients with lung cancer with 1 of 3 emotional personas: depressed, anxious, and frustrated. In total, 30 patient bots (10 per persona) were created, with each provider bot engaging in 30 interactions. The patient bots’ responses were unique due to GPT-4’s stochastic generation processes, even with consistent prompts.

Evaluator bots were created for each provider bot to assess their performance based on predefined criteria, offering scores and qualitative feedback. These AI-generated evaluations were subsequently reviewed by human experts in relevant fields to ensure validity.

Once the evaluator bots reviewed each provider-patient transcript, human experts in each field reviewed the transcripts, scored the interaction using the same criteria as the GPT-4 bots, commented on the provider’s overall performance, and then reviewed the evaluator bot’s assessment.

**Figure 2.**
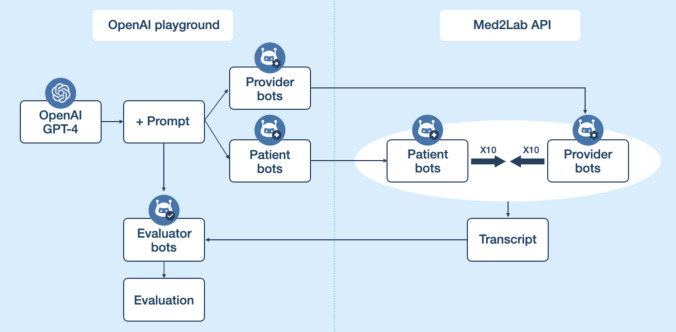
Outline of the bot-bot interactions and evaluations. API: application programming interface.

The patient-education bot was reviewed by the same pediatric hematology-oncology nurse who helped create the patient-education approach, while the pretherapy screening bot was reviewed by a PhD in IT psychology as well as by the cognitive behavior counselor (Figure 2).

### Prompting

**Figure 3.**
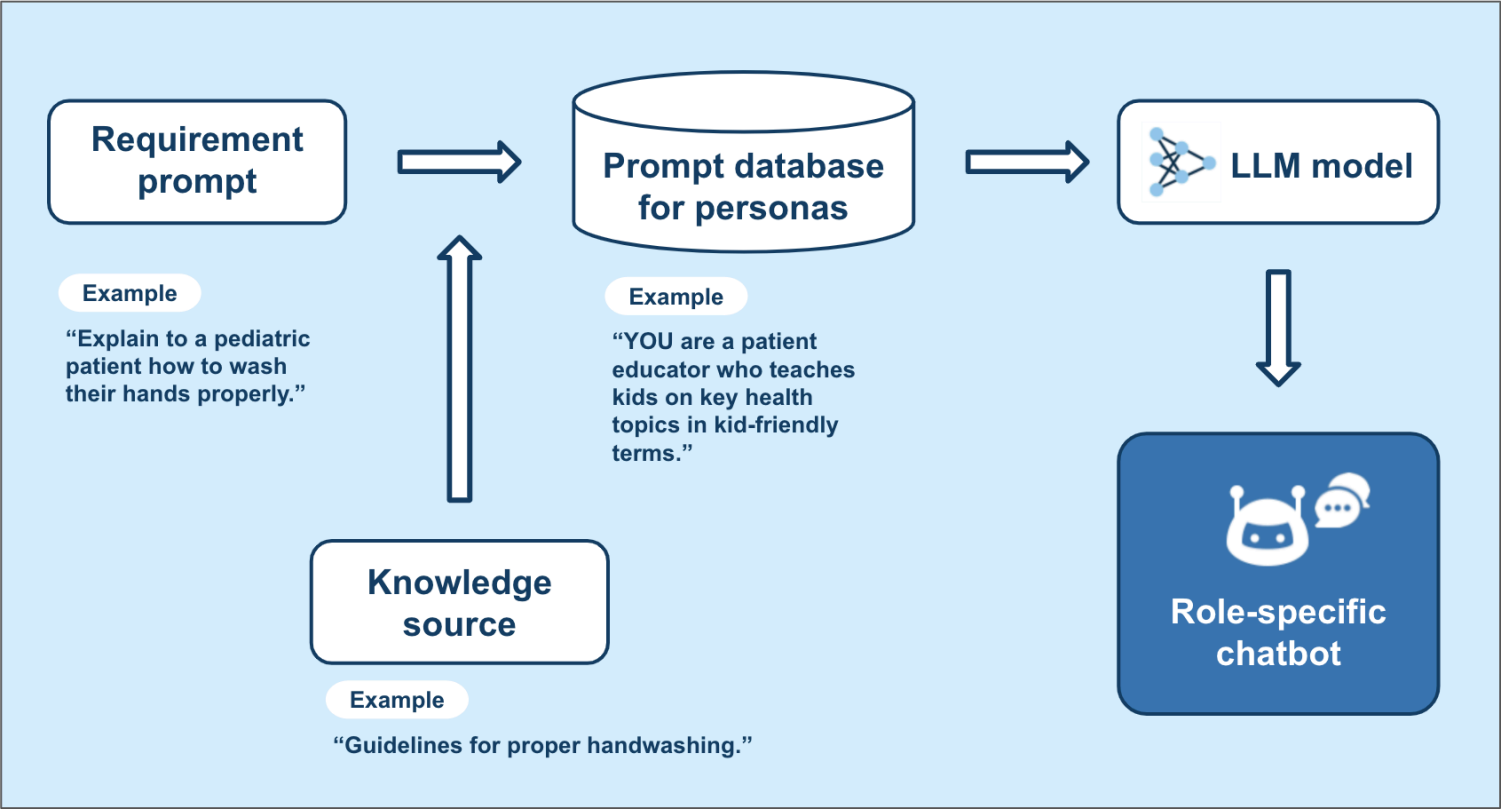
Chatbot architecture. LLM: large language model.

To ensure that the bots adhered strictly to their designated roles while mitigating unwanted behaviors, explicit, role-specific instructions were incorporated into their prompts. This design approach balanced general AI capabilities with task-specific requirements, ensuring consistent and contextually appropriate responses (Figure 3).

#### Patient-Education Bot Prompting

The patient-education bot was designed to emulate the role of a “patient-education nurse” tasked with educating patients with cancer about medical vocabulary, procedures, and treatment options. The bot was prompted with detailed instructions, emphasizing clarity, empathic expressions in communication, and a patient-centered approach. The following guidelines were incorporated into its prompt: (1) adopt a teaching role tailored to patients with limited medical knowledge; (2) provide accurate, comprehensive explanations of medical terms and procedures in simple, relatable language; (3) exhibit empathy and warmth while refraining from making medical recommendations outside the scope of a patient-education nurse; and (4) ensure consistency in tone and responsiveness to patient questions while maintaining a clear boundary of professional role.

#### Screening Bot Prompting

The screening bot was designed to act as a “therapist” specializing in supporting patients with cancer dealing with fear, anxiety, depression, or other stress-related conditions. The prompt emphasized its role in fostering emotional well-being and therapeutic rapport. Key instructions included (1) respond as a therapist practicing nonjudgmental support, inspired by principles from acceptance and commitment therapy and mindfulness practices; (2) reduce patient stress by validating emotions, exploring coping mechanisms, and encouraging hope for change; (3) avoid clinical diagnostic language or prescribing treatments, focusing instead on promoting self-reflection and stress management strategies; and (4) engage the patient through open-ended questions and supportive dialogue, tailored to the specific emotional state of the patient persona.

#### Patient Bot Prompting

The patient bots were modeled to represent 3 distinct emotional personas—anxious, depressed, and frustrated—and were designed to simulate real-life patient interactions. Each patient bot was assigned the role of a 40-year-old male patient with lung cancer undergoing treatment. Detailed persona-specific instructions were included to guide their interactions:

- Persona-specific emotional states:
  - Anxious persona: Expresses uncertainty and seeks detailed explanations.
  - Depressed persona: Exhibits low engagement and responds with shorter, less optimistic answers.
  - Frustrated persona: Displays irritability and impatience in responses.
- Respond consistently with the designated persona’s characteristics throughout the dialogue.
- Do not use or understand high-level medical terms unless explicitly explained by the provider bot.
- For the patient-education bot interactions:
  - Frame responses as questions about unclear cancer-related terms, procedures, or treatments.
- For the screening bot interactions:
  - Actively participate in therapy sessions, responding to the therapist bot’s efforts to reduce stress while maintaining the persona’s emotional tone.

#### Evaluator Bot Prompting

Evaluator bots were designed to act as “supervisors,” assessing the interactions between a provider and a patient. They evaluated transcripts based on a scoring scale (1=poor and 3=excellent) tailored to the respective provider bot’s role.

For the patient-education bot, the following five criteria (maximum score: 15) were used: (1) medical information accuracy, (2) clarity and simplicity of explanations, (3) expressions of empathy and warmth, (4) explanation of purpose or importance of procedures, and (5) adherence to professional role boundaries.

For the screening bot, fourteen criteria (adapted from the Enhancing Assessment of Common Therapeutic Factors tool [12]; maximum score: 42) were used: (1) verbal communication: open-ended questions, summarization, and clarification; (2) relationship building; (3) exploration and normalization of emotions; (4) expressions of empathy and warmth; (5) assessment of functioning and life evaluation; (6) exploration of social support; (7) incorporation of coping mechanisms; (8) evaluation of recent life events; (9) assessment of mental health; (10) collaborative goal-setting; (11) promotion of realistic hope for change; (12) use of simple, jargon-free language; (13) problem-solving steps and processes; and (14) integration of feedback.

Criteria unsuitable for chatbot interactions, such as nonverbal communication, were excluded with detailed reasons listed in Table 1.

**Table 1.** List of Enhancing Assessment of Common Therapeutic Factors (ENACT) factors removed with the reason for their removal.

ENACT factor	Reason for removal
Nonverbal communication and active listening	Therapist is a chatbot and therefore cannot display body language.
Therapist self-disclosure	Therapist is a chatbot and therefore has no real experiences to disclose.
Alcohol or drug and physical problems	Patient has cancer; therefore, physical, alcohol, or drug issues would need to go through their oncologist.
Involvement of family members or caregivers	Patient and therapist are chatbots; therefore, all sessions are assumed to be individual and one-on-one with no family involvement.
Confidentiality promotion	Therapist and patients are chatbots, so all conversations are assumed to be confidential and private.
Assessment of harm to self, harm to others, developing a collaborative response plan	For this study, patient chatbots were assumed not to have violent or suicidal tendencies.

### Provider-Patient Interactions

Each provider bot engaged in 30 unique conversations, distributed evenly across 3 patient personas: anxious, depressed, and frustrated (10 conversations per persona). Conversations were facilitated through an application programming interface designed to streamline the flow of interactions. Each conversation consisted of 20 interactions, defined as 10 turns exchanged between the provider bot and the patient bot.

To simulate concise and realistic clinical exchanges, both provider and patient bots were programmed with the following parameters: a temperature setting of 0.7 (to ensure balanced creativity and consistency), Top P: 1, frequency penalty: 0, and presence penalty: 0. The token limit was removed to avoid interruptions, and each conversation was capped at 10 conversational turns to maintain brevity and clinical relevance. The resulting transcripts from these conversations were reviewed by evaluator bots using predefined criteria and subsequently cross-validated by human experts to ensure the reliability and validity of the evaluations.

### Provider Bot Validation

A 2-step validation process was conducted. First, evaluator bots assessed the provider bots based on predefined criteria, generating scores and qualitative feedback. These results were then reviewed by human experts, a pediatric oncology nurse for the patient-education bot and a cognitive behavior counselor and PhD in IT psychology for the screening bot, using the same criteria used by the evaluator bots to ensure consistency and reliability.

### Evaluator Bot Validation

Evaluator bots graded each interaction transcript based on predefined criteria, producing quantitative scores and qualitative comments. Human experts then reviewed the same transcripts, blind to the evaluator bot’s results, and provided their own scores for comparison. The experts then reviewed the bots’ evaluations to ensure that a consistent and reliable evaluation was carried out by the evaluator bot.

### Statistical Analysis

Descriptive analyses were performed to evaluate interaction characteristics, including word count and sentiment trends. Cronbach α analysis was used to assess the reliability of evaluation criteria across evaluators. Differences in responses between GPT evaluators and human experts were analyzed using the Kruskal-Wallis test. ANOVA was used to identify significant variations in provider bot responses to different patient personas. All analyses were conducted using SPSS (version 24.0; IBM Corp).

### Ethical Considerations

We did not have any human participants or animal subjects and therefore did not need to go before an ethics board.

## Results

### Evaluation of the Patient-Education Bots by AI and Human Evaluators

The patient-education bot, evaluated by both AI and human evaluators, exhibited remarkably consistent performance across interactions with patient bots displaying frustrated, depressed, and anxious personas. The patient-education bot consistently provided accurate medical information, as validated by an experienced oncology nurse, and delivered clear explanations that were fully understood by patient bots, with no instances of confusion reported. Specifically, the AI evaluator assigned perfect or near-perfect mean scores of 15 (SD 0.00), 14.9 (SD 0.31), and 15 (SD 0.00), respectively, while the human evaluator echoed these assessments with similarly high mean scores of 14.9 (SD 0.31), 14.9 (SD 0.31), and 15 (SD 0.00), respectively. The AI evaluator described the patient-education bot to have “... demonstrated excellent skills in providing education and support to the patient. The information provided was accurate, comprehensive, and clearly articulated, catering to the patient’s understanding. The nurse exhibited great empathy and warmth throughout the interactions, which significantly contributed to patient comfort, trust, and engagement. The nurse did not overstep their boundaries by making specific medical recommendations, respecting the role of the patient’s treatment team. Overall, the nurse demonstrated exceptional patient-education skills.”

A singular point of contention arose from the AI evaluator’s interpretation of the patient-education bot potentially recommending treatments beyond its scope. The AI evaluator stated that the nurse could benefit from “being cautious and mindful to avoid being perceived as providing personalized treatment suggestions.” This was later clarified as a misunderstanding, attributing the issue to the AI evaluator’s scoring framework rather than the patient-education bot’s performance.

The patient-education bot was described by the human evaluator to be “correct” and “well-organized and explained,” but the bot’s “[constant expression] of empathy” was reported to “[feel] a bit mechanical.” It was noted that this bot would “likely be helpful, as it can repeatedly explain medical concepts on behalf of medical staff members who do not always have enough time for explanations.”

### Evaluation of the Screening Bot by AI and Human Evaluators

The average AI evaluator bot’s scores for the pretherapy screening bot when interacting with the frustrated, depressed, and anxious patient bots, respectively, were 40.1 (SD 1.28), 40.3 (SD 1.05), and 40.7 (SD 1.15), of a total possible score of 42. Across all 3 patient bots, the lowest scoring criterion was the evaluation of realistic hope for change, which had an average score of 2.53 out of 3 (SD 0.51). Human expert evaluators corroborated the AI evaluation results. The average human evaluator scores of the screening bot when interacting with the frustrated, depressed, and anxious patient bots, respectively, were 37.5 (SD 0.84), 37.6 (SD 0.96), and 36.9 (SD 2.60) for the first reviewer and 36.8 (SD 1.31), 36.9 (SD 1.10), and 36.2 (SD 2.09) for the second reviewer.

The AI evaluator, under the impression it was assessing a human, reported that the pretherapy screening bot excelled in maintaining effective communication, building a warm relationship, and demonstrating empathy. The evaluator bot identified several strengths of the screening bot, stating that it “... provides a warm and empathetic attitude and responds likewise to the patient’s negative reactions and feelings and leads the conversation naturally.” The most common areas for improvement mentioned in the final comments were “exploration of prior successful coping strategies and providing more explicit encouragement for feedback.”

Human evaluators similarly concluded that the pretherapy screening bot excelled in “... [communicating] clearly,” building a “warm and empathetic” relationship, and “[leading] the conversation naturally.” The screening bot reportedly could improve upon “exploring prior coping strategies and patient history a little more deeply” and was occasionally reported to be too informational or talkative. It was reported to “[pass] to the next topic too quickly (possibly due to its large list of duties—which the therapist was prompted to do).” Overall, the human reviewers suggested that “the bot is useful for initial consultations—the AI fluently checks for components of the initial step of counseling.” Furthermore, it was noted that “a more detailed score standard is required for the evaluator bot’s prompt.”

### Statistical Analysis Result

#### Patient-Education Bot

For the patient-education bot, the evaluation scores from both AI and human evaluators were remarkably consistent, showing minimal variance. This uniformity limited the opportunity for further analysis, as the lack of significant differences between evaluator scores precluded more detailed statistical comparisons.

#### Screening Bot

The Kaiser-Meyer-Olkin and Bartlett sphericity test results indicate that the Kaiser-Meyer-Olkin value of 0.714 suggests that the sample is suitable for factor analysis, and the significance probability of Bartlett sphericity test is less than .001, indicating that the correlation between variables is significant. The results of the communality analysis show that all variables have a communality of 1, indicating that all variables explain the extracted factors well. The 5 extracted factors explain 66.327% of the total variance of the variables.

Significant findings from the ANOVA analysis indicate notable variations in group responses across several key evaluation criteria for the screening bot. This variability suggests that specific factors or treatments have a meaningful impact on participant responses, reflecting their efficacy or relevance in different contexts. Verbal communication (open-ended questions, summarization, and clarification) demonstrated a highly significant difference between groups (*P*<.001), suggesting that the approach to verbal communication significantly affects the responses. Assessment of functioning and life evaluation exhibited one of the highest significances (*P*<.001), pointing to the critical role this factor plays in differentiating responses among groups. Exploration of the patient’s social support network also showed a highly significant difference (*P*<.001), indicating a strong effect of social support exploration on participant responses. Assessment of mental health highlighted the most substantial difference between group means (*P*<.001), underscoring the importance of mental health assessment in eliciting varied responses. Evaluation of recent events in the patient’s life and evaluation of realistic hope for change both showed significant differences between groups (*P*<.001 for the former and *P*<.001 for the latter), suggesting these areas notably influence responses.

Other significant areas include relationship building and exploration, interpretation, and normalization of emotions, with *P* values of .004 and .002, respectively, indicating noticeable effects on the responses, albeit less pronounced compared to the areas mentioned earlier. Nonsignificant findings were observed in the expression of empathy, warmth, and genuineness and collaborative goal-setting and managing patient’s expectations, with *P* values of .36 and .28, respectively. These results suggest that variations in group responses to these criteria might not be significantly influenced by the tested factors, potentially due to inherent similarities in the implementation or perception of these aspects.

The use of easy-to-understand vocabulary and integration of feedback, giving advice, and recommendations showed moderate significant differences (*P*=.04 and *P*=.02, respectively), indicating that these areas have a discernible but varied impact on participant responses.

The detailed ANOVA analysis underscores the nuanced impact of different therapeutic communication and evaluation strategies on participant responses. It highlights the areas where specific approaches significantly influence outcomes, offering insights into the effectiveness of various therapeutic and communicative techniques.

The ANOVA results highlight the variability in how different groups responded to the questions. Significant *P* values (*P*<.05) indicate that the groups do not share the same mean response to a question, suggesting that the factor or treatment being tested influences the responses. The strength of this effect varies among the questions, as evidenced by the range of *F* values and *P* values.

## Discussion

### Principal Findings

Overall, the insights gained from this research suggest that AI health care chatbots can be developed, tested, and validated within a relatively short time frame using the 3-bot system. The results of the 3-bot evaluation system suggest that this method can prove valuable for extensive testing of early-stage health care chatbots. The patient bots are able to mimic patient dialogue and provide a platform for the provider bots to output their responses, while the evaluator bot is able to comb through the interaction transcripts and flag any potentially inappropriate responses, greatly reducing the amount of work for researchers. Furthermore, this 3-bot system is highly customizable and can be adapted to fit the needs and cultural norms required by the developers. It is also highly scalable, as the basic requirements to perform the 3-bot evaluations are a computer system and access to an LLM. Performing more iterations of an evaluation only requires a marginal amount of researcher effort, and performing multiple, different evaluations can be accomplished simultaneously, given the computer system has enough processing power.

This study introduces a novel AI-powered health care chatbot validation system featuring 3 types of AI bots—provider, patient, and evaluator. This 3-bot AI system represents a novel methodology not previously explored in existing literature, extending beyond the importance of validation discussed by Bohr and Memarzadeh [2] in AI’s rise in health care, which did not delve into the conversational capabilities between different AI systems in clinical simulations. To our knowledge, our method of testing and evaluating the performance of AI health care provider bots by having them interact with other patient bots and then reviewing the transcripts with an evaluator AI bot has never been reported before.

In our study, we created 2 health care provider bots as examples to demonstrate our system, a patient-education bot and a mental health screening bot. The provider bots were intricately designed to replicate the roles traditionally held by human health care providers, addressing the urgent need for scalable and effective patient care solutions highlighted by Patel et al [13]. These bots are intended to support the health care workforce, which, according to the World Health Organization, is expected to face a significant shortfall [14]. By automating routine tasks, these AI systems could alleviate some of the burdens placed on human staff, allowing them to focus on more complex and sensitive care activities. Already, several health care chatbots are in development, including those designed to answer patient questions and provide mental health therapy [14,15].

However, provider chatbots such as these still require extensive testing, traditionally done by enrolling patients as subjects, which negatively affects the speed and resource cost of developing these tools while running the risk of exposing the patients to unvalidated AI. Therefore, we created 3 types of AI patient bots with personas as examples to test our provider bots. In designing the patient bots, we drew inspiration from Fortin et al [16], who emphasize the importance of personalized and empathetic care in treatment outcomes. In previous studies, various digital patient bots were reported in medical education. In our study, the patient bots were imbued with diverse emotional and psychological states to test the adaptability and responsiveness of the provider bots in a controlled, yet realistic environment, simulating real-life patient interactions.

### Comparison to Prior Work

These current methods require great human input during the iterative testing and evaluation phases, which requires researchers and developers to invest significant time and effort. In contrast, using the 3-bot validation method removes the need for separate human responders and human evaluators, greatly streamlining the initial testing and evaluation process and focusing work efforts on areas of the evaluated bot that require improvement.

Until now, bot-bot interactions were manually reviewed by human experts, which greatly slows the validation process. Current methods of evaluating health care chatbots include a human reviewer reviewing the health care chatbot’s performance against a grading standard as seen in Lechien et al [17] and Goodman et al [18], a human reviewer grading the health care chatbot’s performance against another pre-established chatbot as seen in Aljamaan et al [19], or a mix of the methods, as seen in Huang et al [20].

In this study, we created 2 AI evaluator bots to demonstrate the feasibility of using them as first-line evaluators in addition to human experts. The role of the evaluator bots was crucial in objectively assessing the quality of interactions between provider and patient bots, ensuring adherence to predefined criteria. This evaluation process mirrors the necessity of validation for AI systems before clinical application as emphasized by Kretzschmar et al [21]. By comparing the evaluations conducted by AI evaluator bots with assessments from human experts, we ensured the feasibility of our system, further grounding the study in rigorous scientific methodology. To date, AI bots have been used to review text messages and academic manuscripts, but this is the first study to review dialogue between 2 bots for the purposes of evaluation.

### Limitations and Future Directions

While promising, this study has limitations that warrant consideration. First, the evaluation criteria used were relatively limited in scope, which may not have captured subtle differences in performance between AI and human evaluators. Future research should incorporate more comprehensive and granular criteria to enable more nuanced evaluations. Retrieval-augmented generation could further enhance the evaluator bots by enabling them to cross-verify provider bot responses against dynamic, vetted information sources, thereby increasing the accuracy and reliability of evaluations.

Second, the patient bots were prompted using relatively concise instructions due to the practical constraints of maintaining response speed. This may have limited the complexity and variability of their responses, potentially underrepresenting the breadth of emotions and behaviors seen in real-world patients. Future studies should explore more elaborate prompting strategies or advanced techniques like retrieval-augmented generation to overcome this limitation.

Third, the study used prioritized examples of clinically relevant patient personas (anxious, depressed, and frustrated), chosen for their significance in addressing common and challenging scenarios in clinical practice. While these personas are a high priority for an initial evaluation, they do not fully represent the diversity of patient interactions.

Finally, biases inherent in LLMs may have influenced the results, despite efforts to standardize demographic inputs across all patient bots. Nonessential demographic details were excluded to minimize biases related to race, political affiliation, sexual orientation, or socioeconomic status. Nonetheless, future research should explore the use of specialized LLMs with controlled training datasets to further mitigate such biases.

### Conclusions

We underscore the successful development and implementation of a novel 3-bot evaluation system. This system, consisting of provider bots, patient bots, and evaluator bots, represents a pioneering approach to testing and validating AI functionalities without the need for real patient interactions. Our findings offer a practical solution and set a benchmark for future AI-driven health care services, providing a direction for subsequent research and development efforts.

## Abbreviations

AI: artificial intelligence
LLM: large language model
